# Coexistence of Hodgkin's Lymphoma and Castleman's Disease: A Case Report with Successful Response to Chemotherapy and Radiotherapy

**DOI:** 10.1155/2013/487205

**Published:** 2013-12-05

**Authors:** Amina Mohtaram, Mohammed Afif, Tanae Sghiri, Amal Rami, Rachida Latib, Fouad Kettani, Meryam Ben Ameur El Youbi, Saber Boutayeb, Tayeb Kebdani, Noureddine Benjaafar, Imane Aaribi, Hassan Errihani

**Affiliations:** ^1^Department of Medical Oncology, National Institute of Oncology, Rabat, Morocco; ^2^Department of Radiotherapy, National Institute of Oncology, Rabat, Morocco; ^3^Department of Radiology, National Institute of Oncology, Rabat, Morocco; ^4^Center of Pathology United Nations, Rabat, Morocco

## Abstract

*Background*. Castleman's disease is a rare clinicopathological entity of unknown origin. Coexistence of Hodgkin's lymphoma and Castleman's disease is rare. We report a case of Hodgkin's disease of cervical lymph nodes in a patient previously diagnosed with Castleman's disease. *Case Presentation*. A 43-year-old man admitted in July 2009 for a right cervical pain with lymph node at the physician examination. He underwent a right adenectomy and histological studies showed typical features of Castleman's disease. Three years after, the patient consulted for increasing the volume of cervical lymph node. Clinical examination showed a right cervical lymph node of 3 cm. The computed tomography scan of chest, abdominal and pelvic was normal. Histological and immunohistochemical studies of cervical lymph node biopsy specimen were in favor of Castleman's disease associated with Hodgkin's disease. Reed-Sternberg cells were positive for CD15 and CD30. The patient received chemotherapy based on anthracyclines, bleomycin, vinblastine, and dacarbazine (ABVD) and radiotherapy with complete response. *Conclusion*. Prevalence of Hodgkin's lymphoma in Castleman's disease is more difficult to establish because of the low number of cases reported in the literature.

## 1. Introduction

Castleman's disease also called angiofollicular lymph node hyperplasia, giant lymph node hyperplasia, or angiomatous lymphoid hamartoma is a nonneoplastic heterogeneous lymphoproliferative disorder. It can be classified into a localized form or as a multicentric disease with systemic symptoms. The association between Hodgkin's lymphoma and Castleman's disease is rare and has been well documented. The cases reported in the literature of Hodgkin's lymphoma frequently were of the interfollicular subtype and coexisted with multicentric plasma cell variant of Castleman's disease [1]. We report here a case of a patient diagnosed initially as an unicentric plasma cell variant of Castleman's disease, who later developed an interfollicular Hodgkin's lymphoma on lymph node biopsy with HHV-8-negative morphology.

## 2. Case Report

A 43-year-old man consulted in July 2009 for a neck pain with a solitary right cervical lymph node without general symptoms. He underwent a right adenectomy. Histological and immunohistochemical examinations concluded for features of plasma cell variant of Castleman's disease with cells expressing CD3 and CD20 without expression of CD30 and CD15.

**Figure 1:**
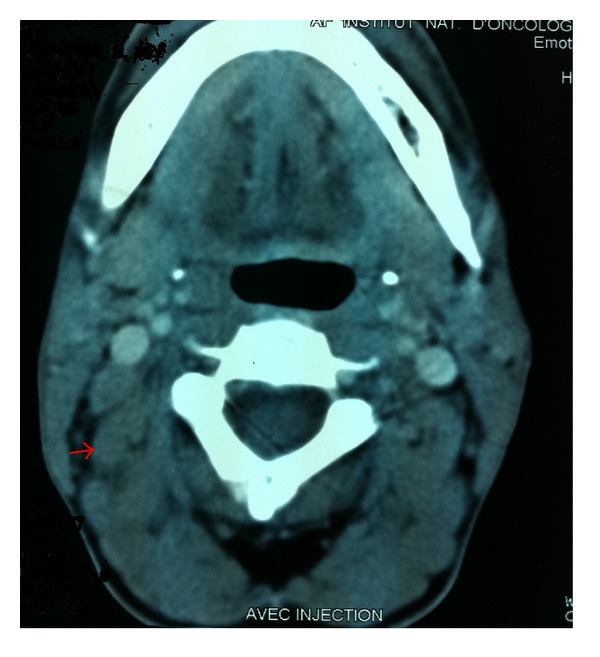
Computed tomography scan of the cervical showed a right cervical lymphadenopathy measuring 3 × 3 cm.

In August 2011 the patient presented an increase in the volume of cervical lymph node without fever or night sweats. Clinical examination revealed two cervical lymph nodes measuring 3 cm and 1 cm without organomegaly. Computerized tomography scan of cervical showed right cervical lymphadenopathy (Figure 1). The chest, abdominal, and pelvic levels were normal. Thereafter, the patient had a cervical lymph node excision.

**Figure 2:**
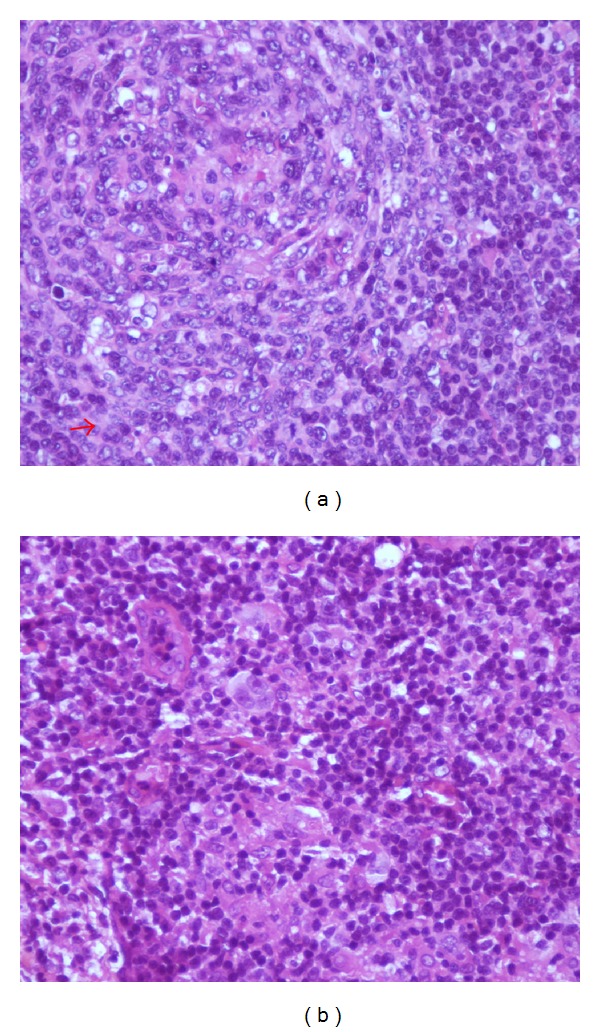
Microscopic findings of lymph node biopsy revealing (a) a preserved lymph node architecture with multiple lymphoid follicles, with or without hyalinized arteriolar (HESx40) and (b) typical Reed-Sternberg cells in the interfollicular.

Histological study of biopsy specimen showed that lymph node architecture is partially normal with presence of multiple lymphoid follicles. The Interfollicular tissue appears homogenized with a polymorphous population of cells composed of lymphocytes, histiocytes, and typical Reed-Sternberg cells (Figure 2).

**Figure 3:**
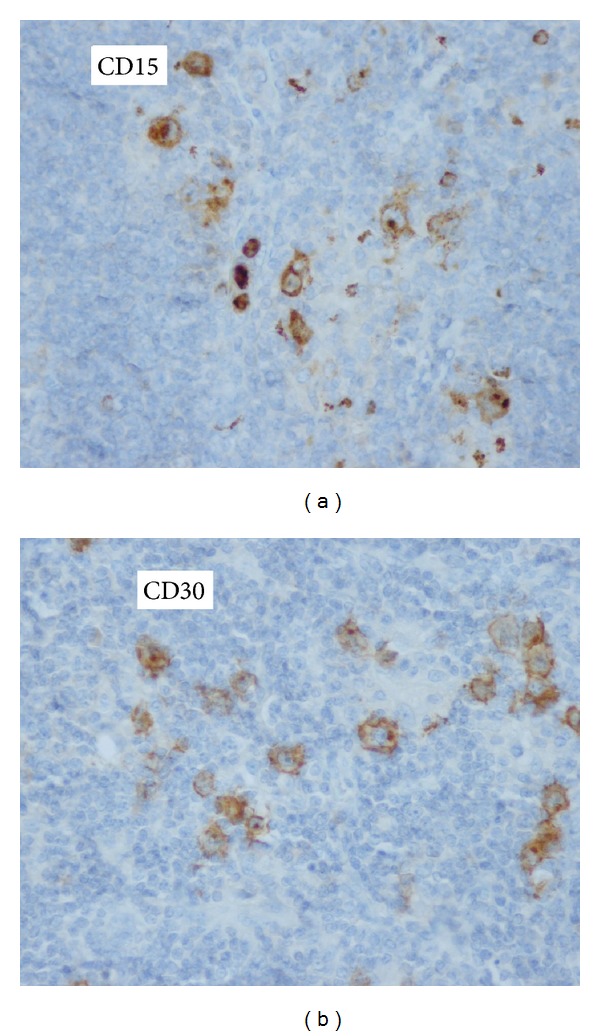
Immunohistochemical analysis showing positive staining of the Reed-Sternberg cells for (a) CD15 and (b) CD30.

In the immunohistochemical study, typical Reed-Sternberg cells were positive for CD15 and CD30 (Figure 3). The anti HHV 8 antibody was negative. Diagnosis finally was in favor of Hodgkin's lymphoma on a background of Castleman's disease.

The bone marrow biopsy was normal. The erythrocyte sedimentation rate was 35 mm and the patient was seronegative for human immunodeficiency virus and for human herpesvirus type 8. Routine laboratory tests were normal.

**Figure 4:**
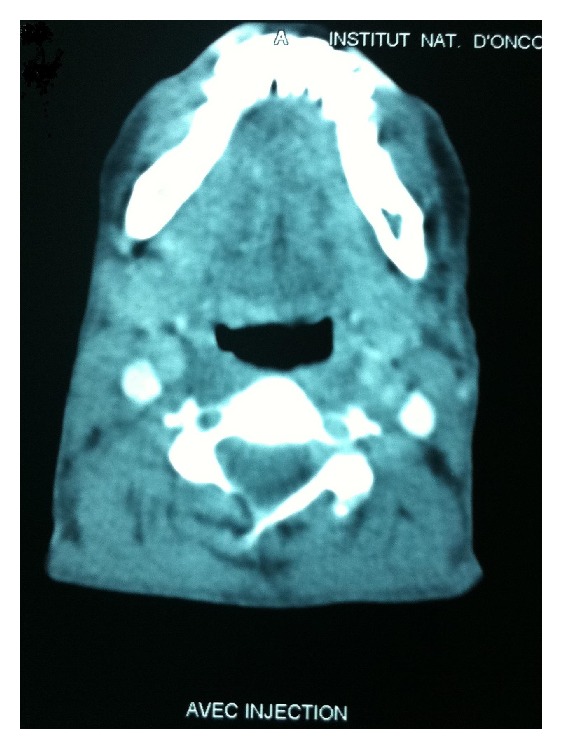
The assessment computed tomography scan of cervical after 4 cycles of chemotherapy showed a complete response.

The patient received four courses of chemotherapy based on the combination of anthracyclines, bleomycin, vinblastine and dacarbazine (ABVD) every 2 weeks. Evaluation by Cheson criteria after 4 courses of chemotherapy was in favor of a complete response (Figure 4). The patient underwent radiotherapy (30 Gy) delivered from March to April 2012 to the involved cervical region.

## 3. Discussion

Castleman's disease is a nonneoplastic lymphoproliferative disorder that occurs throughout the body. Two classification systems exist for this disease: morphologic and histopathogenic classification. The morphologic classification is based on the extent of local lymph node involvement and distinguishes between unicentric and multicentric Castleman's disease [2].

Unicentric Castleman's disease was first described by Benjamin Castleman in 1956. He described 13 patients with hyaline vascular of Castleman's disease in the chest. [3] However, multicentric form was recognized in 1978 and corresponds to systemic disease [4].

Histopathogenetic classification of Castleman's disease includes the classic hyaline vascular type, plasma cell variant, or mixed type. Recent discoveries have classified plasma cell disease into two entities according to the presence of HHV-8 or not [2].

These histological subtypes are important and correlated with clinical syndromes: hyaline vascular Castleman's disease is most often unicentric and usually asymptomatic. If symptoms are present, they are due to a mass effect of bulky lymphadenopathy. Hyaline vascular represents 90% of the cases of Castleman's disease and is more frequently seen in young adults, with a median age in the third or fourth decade [2–5].

Contrary to the hyaline vascular form, plasma cell variant represents less than 10% of Castleman's disease and can be localized (corresponding to 9% and 24% of the cases of localized Castleman's disease) or more frequently multicentric [2]. It is frequently associated with systemic symptoms (such as fevers, night sweats, and hepatosplenomegaly), autoimmune manifestations, recurring infections, and laboratory abnormalities. Plasma cell Castleman's disease can be also associated with the POEMS (polyneuropathy, organomegaly, endocrinopathy, monoclonal gammopathy, and skin changes syndrome) [2–6]. Our case represents an uncentric form of plasma cell variant without constitutional symptoms.

HHV-8-associated Castleman's disease is the plasmablastic variant of Castleman's disease and is found mostly in HIV-positive patients. It is associated with constitutional symptoms, multicentric disease, aggressive course, and likelihood of developing lymphoma when compared to HHV-8-negative cases [7].

Tumor malignancies such as Kaposi's sarcoma, non-hodgkin's lymphoma, and rarely hodgkin's lymphoma have been reported in the course of Castleman's disease [8]. The relationship between Hodgkin's lymphoma and plasma cell-type Castleman's disease has been well documented. After review of the literature, many cases were diagnosed concurrently or were initially diagnosed as plasma cell disease and upon review were found to have interfollicular Hodgkin's lymphoma [9]. In our case histological and immunohistological studies of initial lymph node specimen showed typical Castelman's disease without Hodgkin lymphoma.

The pathogenesis of Castelman's disease is not clear. Some authors hypothesized that cytokines especially interleukin-6 may be involved in causing interfollicular plasmocytosis seen in Castelman's disease due to an increased IL-6 production by the germinal centres [10]. Additionally, human herpesvirus 8 encodes a homolog of interleukin-6 which explains the increase in the systemic levels of IL-6 in Castelman's disease with HHV-8 infection [11]. Recent advances biological basis study showed the roles of IL-1, the RAF pathway, epidermal growth factor receptor, and vascular endothelial growth factor expression in this disease, however, it was illustrated in limited anecdotal cases [12]. These findings have led to the development of promising targeted therapies.

Because Castelman's disease is rare and heterogeneous's makes the standardization of treatment is more difficult. In localized form, the options include excision of the affected lymph nodes and radiation. In multicentric disease, effective therapy requires more extensive treatment based on corticosteroid, radiation therapy, and chemotherapy drugs such as CVP (cyclophosphamide, vincristine, and prednisone), CHOP (cyclophosphamide, doxorubicin, vincristine, and prednisone), or monotherapy (chlorambucil, cyclophosphamide, 2-chlorodeoxyadenosine, carmustine, vincristine, bleomycin, liposomal doxorubicin, oral etoposide, and vinblastine) with varying degrees of response [12].

Targeting HHV-8 replication, CD20, and IL-6 was effective in this disease and durable responses were observed with Rituximab. Tocilizumab is an effective anti-IL-6R antibody that was approved in Japan for treatment of this entity [13].

Siltuximab, an anti-IL-6 antibody, is also effective and is a subject of prospective study in the USA and elsewhere [14, 15]. Other targeted agents were reported in a small number of cases such as the recombinant IL-1R antagonist anakinra, thalidomide, and bortezomib, with success and good tolerance [16–18].

Hodgkin lymphoma occurring within the context of Castleman's disease should be treated with standard lymphoma chemotherapy regimen. Purging of CD20 positive cells with Rituximab might be beneficial by deprived Reed Sternberg cells of a survival factor [6].

In our case, the patient responded well to four courses of ABVD therapy then radiotherapy with complete remission after treatment.

## 4. Conclusion

Castleman's disease is a rare heterogeneous entity with complex physiopathology. It has been associated with lymphoma especially when it is multicentric and associated with human immunodeficiency virus. Coexistence of Hodgkin lymphoma and Castelman's disease is rare and not clear with a few cases reported in the literature. The management therapy for this entity is not standardized when it is confined in the literature to case reports and small case series.
